# An interpretable knee replacement risk assessment system for osteoarthritis patients

**DOI:** 10.1016/j.ocarto.2024.100440

**Published:** 2024-02-16

**Authors:** H.H.T. Li, L.C. Chan, P.K. Chan, C. Wen

**Affiliations:** a Department of Biomedical Engineering, The Hong Kong Polytechnic University, Hong Kong; b Department of Prosthetics and Orthotics, Tuen Mun Hospital, Hong Kong; c Department of Orthopaedics and Traumatology, The University of Hong Kong, Hong Kong; d Research Institute of Smart Ageing, The Hong Kong Polytechnic University, Hong Kong

**Keywords:** Knee osteoarthritis, Machine learning, Prognosis, Self-administrable, Survival analysis

## Abstract

**Objective:**

Knee osteoarthritis (OA) is a complex disease with heterogeneous representations. Although it is modifiable to prevention and early treatment, there still lacks a reliable and accurate prognostic tool. Hence, we aim to develop a quantitative and self-administrable knee replacement (KR) risk stratification system for knee osteoarthritis (KOA) patients with clinical features.

**Method:**

A total of 14 baseline features were extracted from 9592 cases in the Osteoarthritis Initiative (OAI) cohort. A survival model was constructed using the Random Survival Forests algorithm. The prediction performance was evaluated with the concordance index (C-index) and average receiver operating characteristic curve (AUC). A three-class KR risk stratification system was built to differentiate three distinct KR-free survival groups. Thereafter, Shapley Additive Explanations (SHAP) was introduced for model explanation.

**Results:**

KR incidence was accurately predicted by the model with a C-index of 0.770 (±0.0215) and an average AUC of 0.807 (±0.0181) with 14 clinical features. Three distinct survival groups were observed from the ten-point KR risk stratification system with a four-year KR rate of 0.79%, 5.78%, and 16.2% from the low, medium, and high-risk groups respectively. KR is mainly caused by pain medication use, age, surgery history, diabetes, and a high body mass index, as revealed by SHAP.

**Conclusion:**

A self-administrable and interpretable KR survival model was developed, underscoring a KR risk scoring system to stratify KOA patients. It will encourage regular self-assessments within the community and facilitate personalised healthcare for both primary and secondary prevention of KOA.

## 1 Introduction

Osteoarthritis (OA) is the most prevalent joint disease with over 500 million patients globally, over half of them having knee OA (KOA) [1]. It is a multifactorial disease that is influenced by both local and systemic factors, and thus its precise aetiology is elusive, and no cure is available [2]. It has been traditionally conceived as local structural damage due to abnormal mechanical loading exerted on the articular cartilage, which cushions the joint during movement [3]. Nowadays, researchers are placing much emphasis on the contribution of systemic risk factors to the development of KOA, among them, population ageing and obesity pandemic account for a sharp rise in the prevalence of KOA [4]. Also, sedentary living habits, comorbid metabolic syndrome and elevated use of pain medications are also major systemic risk factors for accelerated KOA [[5], [6], [7], [8]].

The course of KOA varies hugely among individuals. Only a few patients progress rapidly to the end-stage OA, which requires surgical replacement, a major operation with significant morbidity and mortality [9]. In contrast, most of the KOA cases are stable under conservative treatment [5]. However, in current clinical path, all the patients are receiving a unified treatment protocol passively regardless of natural course of disease, from health education, conservative treatment including pain medications to joint replacement surgery. As a consequence, the golden window of therapeutic opportunity for the rapidly progressive KOA cases will be missing, increasing patients’ suffering and incurring unnecessary socioeconomic and healthcare costs. On the other hand, our limited healthcare resources will be wrongly allocated to those stable KOA cases. Therefore, it is imperative to triage KOA patients in the early stage of disease, particularly for those at high risk of rapid progression.

Recently, scientists began to adopt Machine Learning (ML) to predict the course of KOA and unveil the conducive and preventive risk factors in an attempt to deliver personalised treatment for better disease management. However, those models incorporated kinematic data derived from gait analysis and imaging data from X-ray and magnetic resonance imaging (MRI), which are deemed impractical in large-scale routine community screening with the high cost and limited accessibility [10,11]. In light of this, other researchers utilised simple predictors such as sociodemographic and anthropometric data, and clinical measurements for KOA prognosis, with the prediction performance up to 0.68 in area under the receiver operating characteristic curve (AUC) [12]. However, it only performed binary classification to predict the occurrence of KOA deterioration. This neglected the time to develop KOA, which contained much more clinical information than the binary outcomes [13]. Thus, a survival analysis model for knee replacement (KR) was devised to estimate the time for KR with an AUC of 0.86 [14]. Nonetheless, radiographs were also used for prediction that impeded its community screening adaptation.

Patient stratification based on development risk should be performed for better primary and secondary preventive strategies in which individuals can be prioritised to receive appropriate treatment to salvage their knee joint. Therefore, in this study, it is prime time to develop an ML-based KOA risk stratification system to achieve the following objectives:

1. To develop a self-administrable, ML-based KOA risk stratification system to predict the risks of KR incidence using easily accessible self-reporting clinical features namely demographics, modifiable lifestyle-related outcomes, systemic conditions, lower limb conditions, and patient-reported outcomes to enable community-wide screening.
2. To employ the model interpretation algorithm for the evaluation of feature importance, providing insights for individualised treatment approaches.

## 2 Method

### 2.1 Data acquisition and exclusion criteria

In this study, the Osteoarthritis Initiative (OAI) dataset from the National Institute of Health Osteoarthritis Biomarkers Consortium was used. There were clinical measurables from 9592 knee samples from their first visit (baseline) through 96 months after the baseline, with a follow-up session every year thereafter. The subjects were between 45 and 79 years old and at risk of developing KOA. Rather than relying on medical images such as X-rays and MRI for prediction, patient characteristics and clinical data were used as prediction features, to incentivise self-administrable community screening. Based on a comprehensive review of KOA pathophysiology from current studies, a total of 14 potential risk factors from subjects’ demographic information, modifiable lifestyle-related outcomes, and co-morbidities collected from the screening visits were investigated [5,[15], [16], [17]] (Table 1). Since structural alterations of KOA become progressively evident typically after 3–4 years or more, this signifies the loss of joint homeostasis with no approved treatment could reverse the progression [16]. Therefore, the prediction outcome for KR incidence was determined by 48 months after the first clinical visit (i.e., the baseline) in this study to potentially encourage susceptible people to receive appropriate treatment promptly to salvage their knee joint.

**Table 1:** Baseline characteristics of the study populations in the Osteoarthritis Initiative (OAI) dataset.

Features/Risk factors	Categories	Total study population (*n* ​= ​9,592 knees)	Train set population (*n* ​= ​7,673 knees)	Test set population (*n* ​= ​1,919 knees)
Sex	0: Male	3984 (41.5%)	3200 (41.7%)	784 (40.9%)
	1: Female	5608 (58.5%)	4473 (58.3%)	1135 (59.1%)
Age, years	Mean (Standard deviation)	60.3 (9.19)	61.1 (9.20)	61.3 (9.13)
Education level	1: Less than high school graduate	336 (3.5%)	275 (3.6%)	61 (3.2%)
	2: High school graduate	1214 (12.7%)	977 (12.7%)	237 (12.4%)
	3: Some college	2311 (24.1%)	1835 (24.0%)	476 (24.8%)
	4: College graduate	2050 (21.4%)	1660 (21.6%)	390 (20.3%)
	5: Some graduate school	809 (8.4%)	654 (8.5%)	155 (8.0%)
	6: Graduate degree	2872 (29.9%)	2272 (29.6%)	600 (31.3%)
Body mass index (BMI), kg/m^2^	Mean (Standard deviation)	28.6 (4.84)	28.6 (4.84)	28.5 (4.83)
Occupational activity level in the last 7 days	1: Sitting	1912 (20.0%)	1522 (19.9%)	390 (20.2%)
	2: Sitting/standing/walking	5635 (58.7%)	4511 (58.8%)	1124 (58.6%)
	3: Walking/handling <50 lbs	1787 (18.6%)	1431 (18.6%)	356 (18.6%)
	4: Walking/handling >50 lbs	258 (2.7%)	209 (2.7%)	49 (2.6%)
Smoking habit	0: No	8946 (93.3%)	7145 (93.1%)	1801 (93.9%)
	1: Yes	646 (6.7%)	528 (6.9%)	118 (6.1%)
Stroke history	0: No	9314 (97.1%)	7452 (97.1%)	1862 (97.0%)
	1: Yes	278 (2.9%)	221 (2.9%)	57 (3.0%)
Diabetes history	0: No	8868 (92.5%)	7097 (92.5%)	1771 (92.3%)
	1: Yes	724 (7.5%)	576 (7.5%)	148 (7.7%)
Heart attack history	0: No	9404 (98.0%)	7518 (98.0%)	1886 (98.3%)
	1: Yes	188 (2.0%)	155 (2.0%)	33 (1.7%)
Use of pain medication	0: No	4285 (44.7%)	3387 (44.1%)	898 (46.8%)
	1: Yes	5307 (55.3%)	4286 (55.9%)	1021 (53.2%)
Knee injury history	0: No	7000 (73.0%)	5627 (73.3%)	1373 (71.5%)
	1: Yes	2592 (27.0%)	2046 (26.7%)	546 (28.5%)
Knee surgery history	0: No	8350 (87.1%)	6684 (87.1%)	1666 (86.8%)
	1: Yes	1242 (12.9%)	989 (12.9%)	253 (13.2%)
Persisting knee pain and stiffness	0: No	2757 (28.7%)	2213 (28.8%)	544 (28.3%)
	1: Yes	6835 (71.3%)	5460 (71.2%)	1375 (71.7%)
Use of walking aids	0: No	9458 (98.6%)	7561 (98.5%)	1897 (98.9%)
	1: Yes	134 (1.4%)	112 (1.5%)	22 (1.1%)

### 2.2 Missing data handling

The missing data in both train and test sets were imputed by Multivariate Imputation with Chain-Equation (MICE) algorithm. This imputation process was performed iteratively, where the missing values of the variables are imputed multiple times, generating a complete dataset [18]. Being a reliable method to obtain missing value estimation, MICE enables accurate measurement of uncertainty in the following statistical analyses.

### 2.3 Survival model selection

The Cox Proportional Hazards (CPH) has been a conventional semiparametric statistical method for survival modelling, which is to calculate the impact of features on the odds of an event happening [19]. The CPH model is most frequently used in the oncology field to identify the impact of different prognostic factors on the patient's disease progression, recurrence and ultimately survival [20,21]. However, one major drawback of the CPH model is its linear function nature that assumes feature independence, which may fall short in more complex analyses when compared with other ML models such as Survival Trees, Gradient Boosting Machine, and Random Survival Forests that account for the nonlinear approximation of the statistical relationship between features [[22], [23], [24]].

In subsequent analyses, four survival models using CPH, Survival Trees, Gradient Boosting Machine, and Random Survival Forests were developed to predict 48-month KR incidence. The dataset was divided into a train and test subset randomly with an 8:2 ratio, which is a heuristic approach commonly adopted by researchers due to the absence of an optimal partitional method [25]. The larger portion was used for training with cross-validation, while the smaller portion remained unseen from the trained model for independent testing. The hyperparameters were tuned based on the concordance index (C-index) using grid-search with 5-fold cross-validation on the train set. To evaluate the classification performance, the model was tested with 1000 iterations of bootstrap sampling, and the means and standard deviations were calculated for the C-index and average AUC. The performance of each model was compared, and the one with the highest average AUC score was selected for subsequent feature selection.

### 2.4 Feature selection

To improve computational efficiency and reduce the generalisation error, a feature selection pipeline was implemented to reduce the initial 14-dimensional feature space to a *k*-dimensional subspace, where *k* ​< ​14. This enabled the identification of the most relevant features. Thus, the most relevant subset of features for the disease outcome prediction was selected by using Sequential Forward Selection (SFS), a feature selection algorithm under the wrapper model category. In contrast to filter-based approaches, another major category of feature selection algorithms which disregards the impact of features selected on the performance of the ML model, the wrapper-based framework could yield a feature subset better tuned to the interactions with the classifier [26]. It is also less prone to overfitting due to the inherent cross-validation procedure during the iterative selection process, leading to a more robust model performance.

SFS was employed in the train set and started with an empty feature subset. There were 14 feature subsets created in the first round of iteration, with each containing only one feature. The survival model was trained from each subset with the training set samples. The prediction performance was evaluated with 5-fold cross-validation with the independent test set to avoid overfitting. Feature subsets with the highest C-index scores were selected for the next iteration. In every iteration, a single feature was added to the feature subset. It was repeated until all 14 features were included in the feature subset. The feature subset that yielded the highest C-index score from the 14 iterations was selected to be the optimal number of features, *k*, and became the reduced model.

### 2.5 Knee replacement risk stratification

KR risk scores were calculated from regression coefficients in the survival model, then rescaled into ten-point scales for analysis. In this KR risk stratification system, subjects were differentiated into three risk levels, with two thresholds being used to determine the likelihood of KOA negative (KOA-), KOA positive (KOA+), and knee replacement incidence (KR+) at 48 months. The higher threshold represented the classification of KR+ patients at the 48-month timepoint, while the lower threshold was designed to predict the 48-month KOA incidence based on the Kellgren-Lawrence (KL) grading system. Specifically, a KOA-subject possessed a KL grade 0 or 1, while a KOA+ subject was confirmed with a KL grade 2 to 4 [27]. Subjects in test set with missing KL grade were therefore omitted. The two thresholds were optimised by maximising the F1-score as a metric used in binary classification in the training cohort. The KR risk stratification performance was evaluated by Kaplan-Meier analysis and the Sankey plot. Separations of the survival curves were compared with the log-rank test.

### 2.5 Survival model interpretation

To interpret the relative contributions of the risk factors, Shapley Additive Explanations (SHAP), was implemented on the survival model. It uses Shapley values from coalitional game theory to represent the impact of each feature on the prediction outcome by averaging marginal contributions to feature values across all possible coalitions [28]. Thus, this accounts for the individual predictions at a given instance. Doctors can use this local explanation for designing treatments tailored to individual patients. The global contribution of each feature can be obtained by averaging the SHAP values across all time points and samples to decipher the aetiology of KOA.

## 3 Results

### 3.1 Model performance

**Fig. 1:**
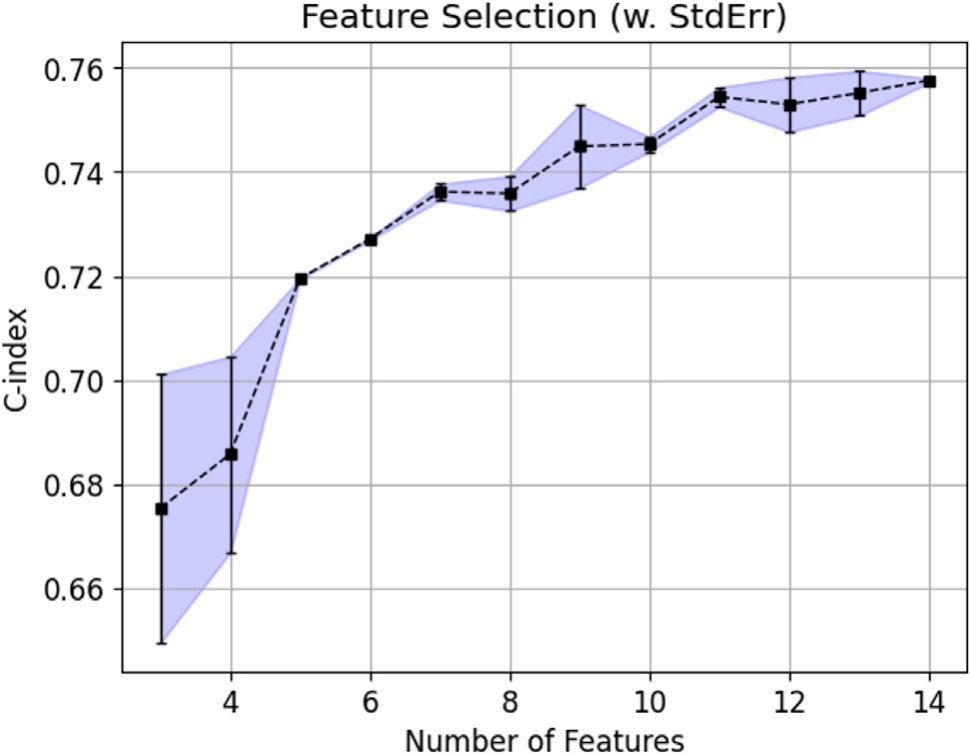
The plot of concordance indices (C-indices) against the number of features derived from Sequential Forward Selection (SFS).

Of all the models evaluated, the Random Survival Forests model with 14 features demonstrated to most accurate prediction of the KR incidence in four years with the highest average AUC of 0.807 (±0.0181) and a C-index of 0.770 (±0.0215) on the independent test set (Table 2). Further feature selection pipeline from SFS did not yield any improvement on the C-index across all feature combinations (Fig. 1).

**Table 2:** Performance comparison of different machine learning models on the test set.

	C-index	Average AUC
Random survival forests	0.770 (±0.0215)	0.807 (±0.0181)
Cox proportional hazards	0.757 (±0.0202)	0.800 (±0.0212)
Gradient boosting machine	0.703 (±0.0372)	0.717 (±0.0150)
Survival tree	0.591 (±0.0196)	0.591 (±0.0267)

### 3.2 Knee replacement risk score

**Fig. 2:**
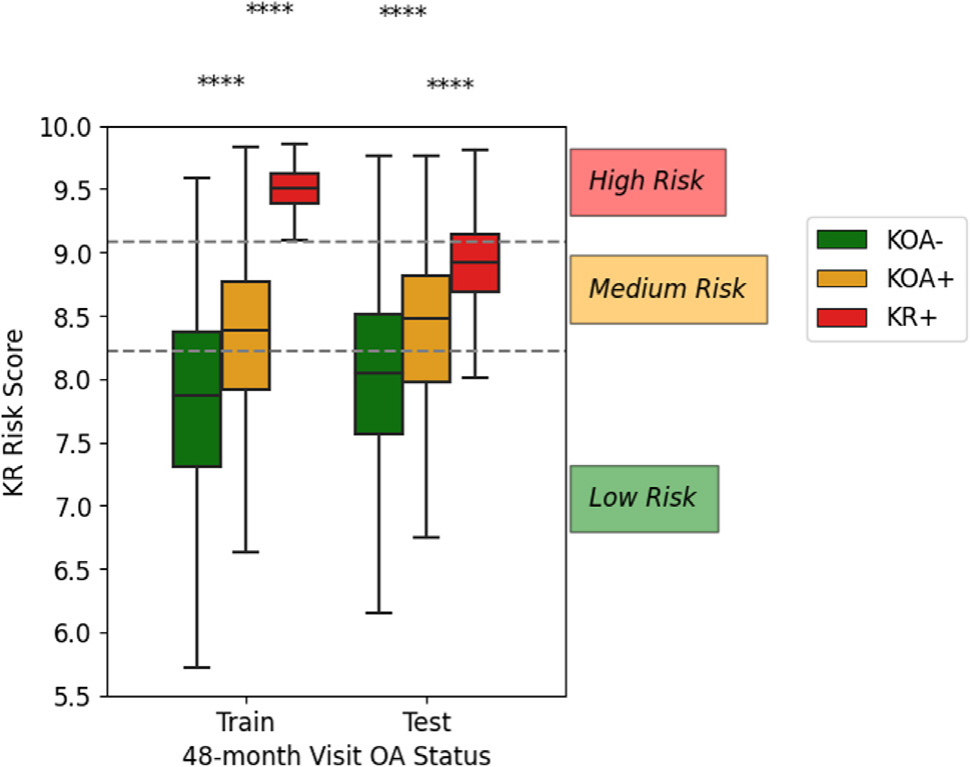
The distribution of knee replacement (KR) risk scores for the three progression endpoints (i.e. KOA-: Knee osteoarthritis negative, KOA+: Knee osteoarthritis positive, and KR+: Knee replacement incidence) at the 48-month follow-up. The two dash lines represented the thresholds of the KR risk stratification system calculated from the training cohort.

**Fig. 3:**
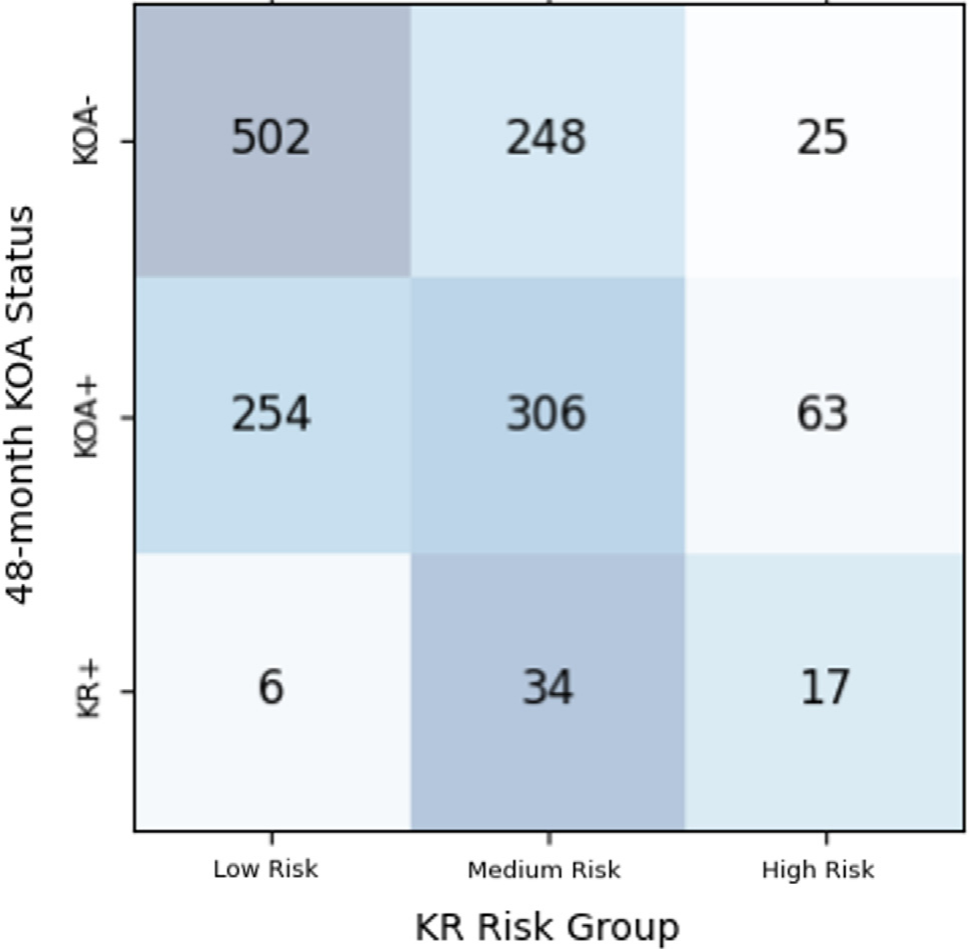
The confusion matrix of the four-year progression classification under the knee replacement (KR) risk stratification system on testing set samples.

Among the 1919 subjects in the test cohort, 464 of them had missing KL grade, with 1455 remaining subjects in test set being eligible to compile the KR risk score. Significant differences in KR risk score were observed among the three KOA development outcomes at the 48-month follow-up (Fig. 2). As derived from the testing cohort, the non-KOA subjects shared the lowest average risk score of 7.50 (±0.755), whereas the incident KOA and KR subjects had a statistically higher average risk score of 8.63 (±0.231) and 9.28 (±0.165) respectively from the Mann–Whitney-*U* test with Bonferroni correction (*p* ​< ​0.001). By maximising the F1-score of the multiclass prediction, the two optimised lower and higher thresholds were calculated as 8.22 (±0.0117) and 9.08 (±0.0328) respectively. Supplemented by the confusion matrix (Fig. 3), a risk score lower than 8.22 were categorised as low-risk group (*n* ​= ​762) with minimum risk of disease development (33.3%) and KR incidence (0.79%). Meanwhile, a risk score between 8.22 and 9.08 was determined as medium-risk group (*n* ​= ​588) with a moderate risk of KOA (52.0%) and KR incidence (5.78%). Lastly, those a score more than 9.08 were considered as high-risk group (*n* ​= ​105), with more than half of the population experiencing KOA incidence (60.0%) and standing the highest chance (16.2%) of receiving a KR surgery promptly.

**Fig. 4:**
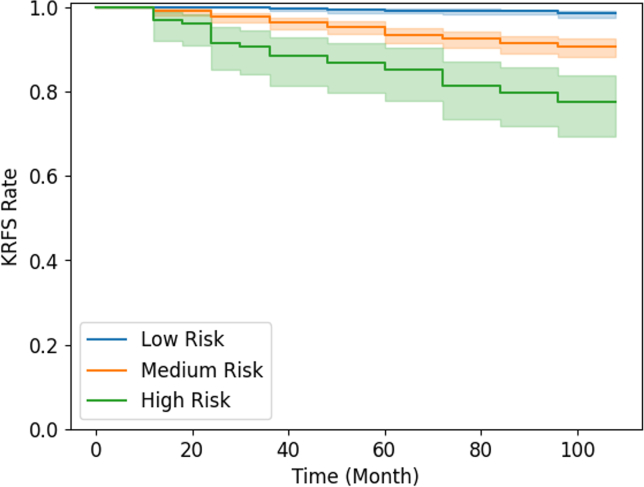
The Kaplan-Meier plot depicted three survival curves indicating the knee replacement (KR)-free survival rate of the three risk groups on the test set.

**Fig. 5:**
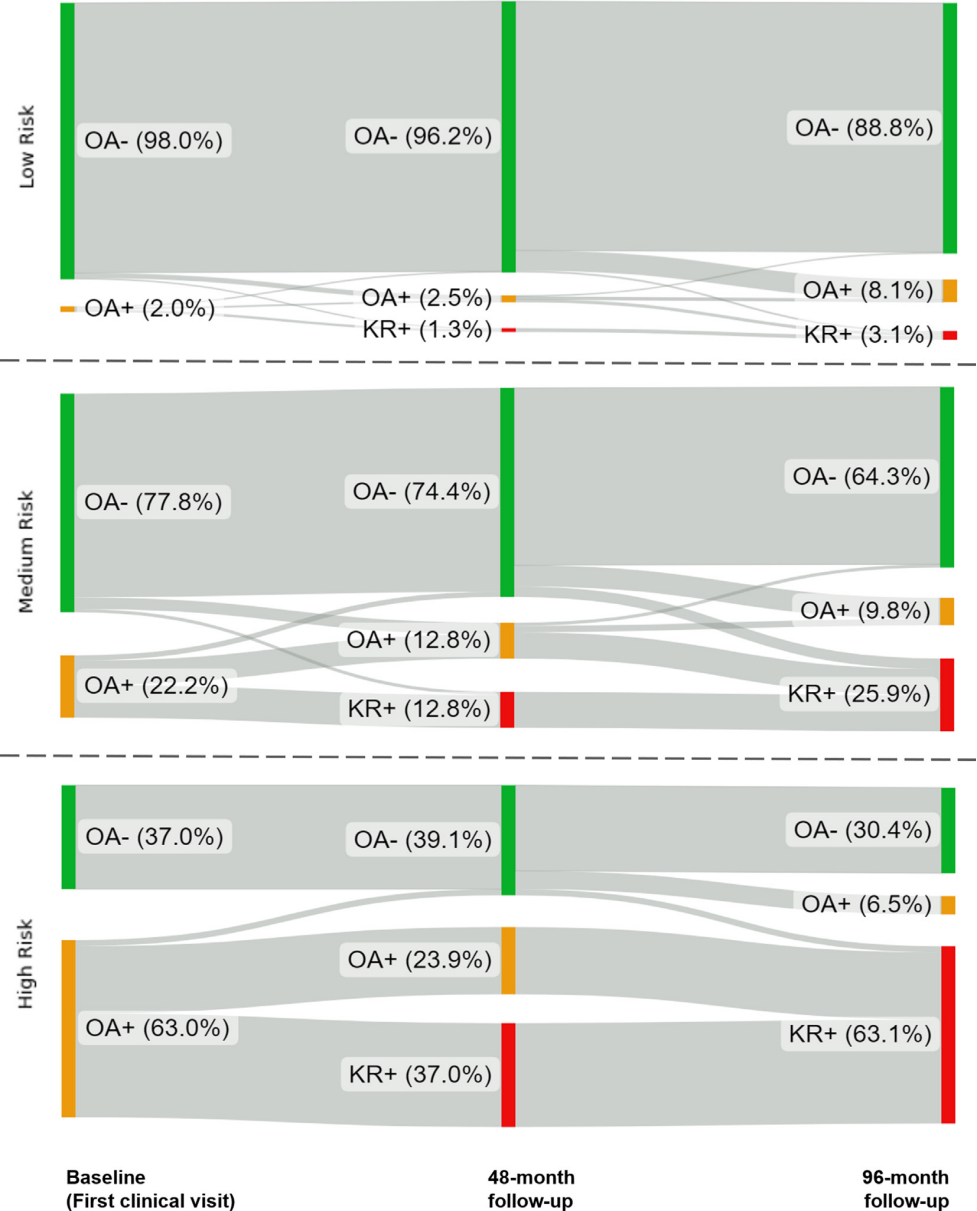
The Sankey plots of the knee osteoarthritis (KOA) progressions in multiple tiers of risk level at baseline, 48-month, and 96-month follow-ups.

Distinct survival patterns among the three risk groups were shown by the Kaplan-Meier plot (Fig. 4). Further log-rank tests discovered significantly different patterns across all survival curve comparisons (*p* ​< ​0.001). To investigate the validity of the KR risk score beyond the four-year timeframe, the Sankey plot visually depicted the development of KOA and KR incidence of the three risk groups at the baseline clinical visit, 48-month, and 96-month follow-ups (Fig. 5). In the low-risk group, the majority of them remain KOA- (88.8%) while only 8.1% and 3.1% of the samples ended with a KOA onset and KR incidence by the end of 96-month respectively. The medium-risk group shared modest odds of disease development as the KOA+ population rose slightly to 9.8% and over a quarter of the samples (25.9%) would eventually require KR in eight years. As a corollary, the KOA+ population declined to 64.3% in the medium-risk cohort. Meanwhile, the proportion of KOA outcomes was drastically dissimilar in the high-risk group compared with the other risk groups. It was noticed that over half of the high-risk samples (63.1%) opted for KR eventually, leaving only 30.4% of them remained KOA free.

### 3.3 Model interpretation

**Fig. 6:**
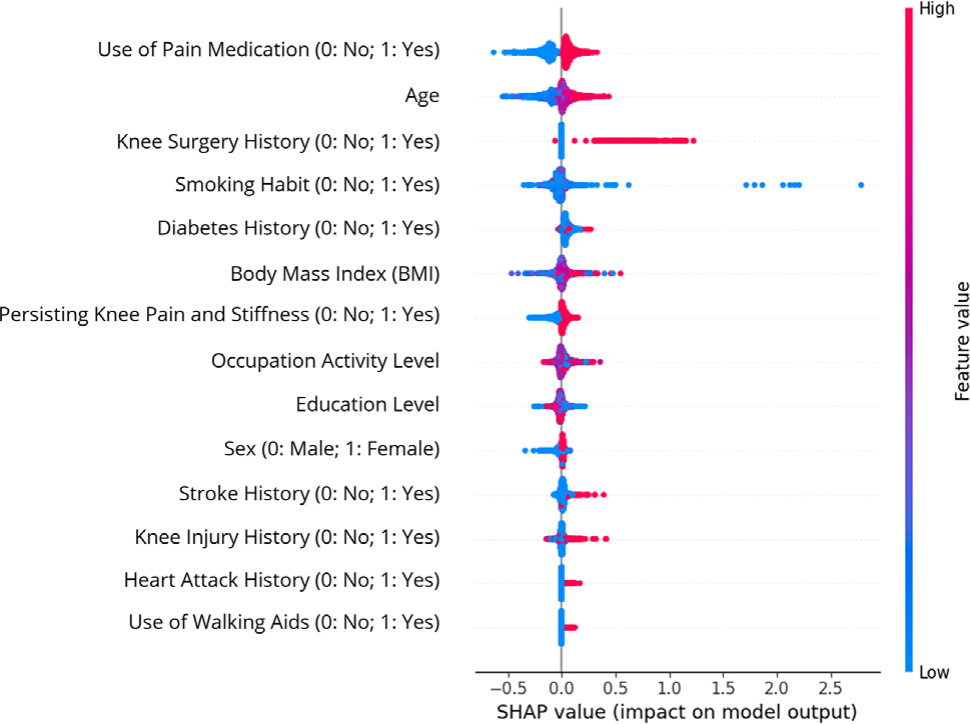
The summary plot of Shapley Additive Explanations (SHAP).

The influence of the predictors on KOA prognosis in the ML model was concluded from the SHAP summary plot (Fig. 6). The vertical axis showed the order of importance of the predictors from top to bottom, whereas the horizontal axis represented the impact on the model classification output with the SHAP value, i.e. a greater importance (SHAP) value indicated the more significant prediction to KR incidence, while the negative value favoured the prediction to the non-KOA class. Meanwhile, each dot from each predictor represented each sample and was coloured according to their feature value, with low value in blue and high value in red. As revealed by the summary plot, the regular use of pain medications, older age, a history of undergoing knee surgery and diabetes, and a high body mass index (BMI), were emerged as the most contributing factors to KR incidence at 48-month follow-up. However, being a non-smoker yielded inconclusive results as blue data points were dispersed in both directions on the SHAP value axis.

## 4 Discussion

### 4.1 Model performance and comparison with similar models

In this study, a ML-driven KR survival model was developed to predict the risk and time to KR incidence within four years using the OAI dataset. Random Survival Forests was employed to capture the non-linearity of in the dataset and potentially confounding interaction between the risk factors. To improve computational efficiency and reduce generalisation error, SFS was implemented thereafter and confirmed that the model with all the 14 features yielded the most robust prediction performance with C-index of 0.770 (±0.0215) and an average AUC of 0.807 (±0.0181) from the independent test cohort. Surprisingly, despite using simple predictors, our performance was comparable to other similar survival model for KR using X-ray (KL grade) and MRI with a C-index of 0.85 and AUC of 0.86 [14]. Although X-ray and MRI have been proven in previous research as the optimal image modality in assessing KOA pathology, their high cost and risk of over-diagnosis may hinder its adaptation in routine clinical examinations [10].

Sharing similar intention to incentivise early disease identification through primary care and self-assessments, the clinical model developed by Liu, Chu, LaValley, Hunter, Zhang, Tao et al. [29], with a C-index of 0.78, and us were intentionally designed to incorporate simple risk factors with comparable prognostic ability in KR incidence. Nonetheless, our strategy held three key advantages in KOA management. For one, the model developed by Liu, Chu [29] was designed for clinicians, while our target audience consists of the elderly and their caregivers. Thus, our model strives to ensure that the assessment process is user-friendly and self-administrable, empowering the user to accurately complete the survey by themselves. We therefore considered each comorbidity separately, whereas it was represented collectively with the Charlson Comorbidity Index in their model. Moreover, their model required the user to correctly identify the use of each medication, while we just consider the use of medication as one variable. Furthermore, the Western Ontario and McMaster Universities Arthritis Index (WOMAC) score employed in their clinical model comprises numerous items, and that could be burdensome for our target users. Hence, we did not include the WOMAC score in our analysis. For two, with a high model interpretability using SHAP, clinicians could devise therapeutic plans that pinpoint patients' own modifiable living habits and health outcomes. With pre-emptive and customised treatments available to vulnerable individuals, their knee health could be salvaged within the “window of opportunity” to prevent irreversible knee damage [16]. For three, since delayed KR would impair treatment outcome, by quantifying the likelihood of receiving KR in a three-tier scoring system, our model could be an intuitive tool for assisting surgical prioritisation independent to doctors’ experience [30].

From the confusion matrix in Fig. 3, there were misclassifications in the model, and thus the baseline characteristics of the misclassified individuals in comparison to their correctly classified counterparts. Concerning the misclassification of KR+ patients as low-risk individuals, we found that a significant proportion of these patients did not use pain medication, unlike the KR+ patients in the high-risk group (*p* ​< ​0.01). It is worth noting that pain is a crucial factor in the decision-making process for KR. Therefore, our model may have treated the lack of pain medication usage as a sign that these patients did not require KR. Meanwhile, the misclassification of KOA-patients as high-risk individuals was associated with several statistically significant factors. These misclassified patients had an older age, higher BMI, and a higher proportion of them reported with the use of pain medication, in addition to having a history of knee injury and surgery, compared to the KOA-patients in the low-risk group (*p* ​< ​0.01). These characteristics are also commonly found in both KOA patients and knee replacement recipients. Hence, it is probable that our model incorrectly considered these individuals as high-risk patients owing to these shared characteristics.

### 4.2 Predictors for knee replacement survival analysis

From SHAP, the regular use of pain medications, older age, and a history of undergoing knee surgery, played the most pivotal role in predicting KR incidence. Although these factors could also be observed at a later stage of KOA, they are playing a vital role towards the development of the disease at the early stage. Aging is one of the factors that drive the vicious cycle of a series of pathophysiological processes of OA [15]. Muscle weakness, joint laxity, and impaired proprioception generally aggravate with age, leading to abnormal biomechanical loading of the knee. It is followed by mechanoflammation and degradation of the knee joint, inducing painful symptoms that deters the participation in physical exercises and the cycle reiterates itself. Besides, individuals who have sustained a knee injury or undergone surgery share a less stable joint structure and become vulnerable targets to secondary OA [5]. Hence, they are at a heightened risk of KOA development and receiving joint replacement at a younger age in comparison with their uninjured counterparts [31]. Lastly, it is suggested that KOA patients may encounter activity-induced knee pain at the early stage, and thus the use of pain medication could indicate early KOA [16].

Our results also suggest the predominant role of the high BMI value as a systemic factor in KOA development and KR incidence. Ample international clinical guidelines advocate weight loss for overweight or obese individuals since they are more prone to KOA onset and deterioration [17]. A weight loss for returning to normal BMI can reduce the mechanical load in the knee joint, thus preserving the joint structure and improving the effects on pain and function [32]. Obesity also induce mechanoflammation of KOA by altering adipokine levels to stimulate the production of proinflammatory factors and degradative enzymes, suppressing cartilage matrix synthesis and leading to subchondral bone remodelling [6]. It has also been argued that OA may be the result of a systemic metabolic disorder, as people with OA are influenced by other weight-related comorbidities such as elevated blood pressure, insulin resistance, and dyslipidemia, which are often discovered in obese persons [33]. Since BMI and diabetes are the few modifiable risk factors in the survival model, our findings may provide future preventive strategies for reducing the odds of KOA development.

Interestingly, the smoking habit has led to conflicting results regarding its role to KOA deterioration and KR incidence. Research has shown that smokers tend to have a more sedentary lifestyle and decreased bone density, accounting for less mechanical stress and thus the wearing of the knee joint [7,34]. Besides, nicotine also binds with nicotinic acetylcholine receptors the enhance the levels of neurotransmitters, thereby promoting an anti-inflammatory effect [35]. Despite these perceived protective effects to the knee joint, it is still not advocated to adopt smoking as a means of KOA prevention given that smoking interacts with the predisposed OA genetics [36]. Moreover, smoking has been shown to be associated with metabolic syndromes, which are linked to KOA deterioration, further compromising knee health [37,38].

### 4.3 Strengths and limitation

The KR risk stratification system could aid in better triage. In contrast to the previous prognostic models for KOA, our survival model could reveal much more information by estimating the time to the KOA onset and KR incidence rather than simply giving out binary outcomes such as logistic regression [13]. It can be implemented in healthcare settings as a web-based programme to enable medical personnel to conduct assessments efficiently using simple clinical predictors. This provides an accurate and intuitive approach to differentiate individuals with various states of disease inertia. Mild patients can receive treatment within the community, whereas high-risk subjects who would experience joint failure rapidly could be prioritised with precise rehabilitation therapy and surgical intervention in secondary healthcare. This would be instrumental to the primary and secondary prevention of KOA, relieving the hefty profound socio-economic burden arising from this debilitating disease [5,39].

Moreover, the model explanatory tool can equip the public with self-management skills. OA education has been recognised as the first and most important step in OA treatment to promote the importance and strategies in OA self-management and coping with the disease [40,41]. This can be achieved by incorporating our survival model in a mobile application through which older adults and their caregivers can perform self-administrable KR risk assessments followed by explained results. This provision of accurate and interpretable health information fosters a sense of self-ownership of their own health in patients and aids in the development of self-management skills [42]. Health education for the elderly is especially crucial because negative beliefs and fallacies about their health conditions may result in worse treatment outcomes [17]. Besides, the transparent model explainability can generate explicit justifications of how the model derives the prediction output, laying a foundation for clinicians to triage and tailor individualised programmes to patients in accordance with patients’ condition and needs for optimised outcomes [43].

There are a few limitations in this study. Although the model has been validated with an independent test set, they are from the same dataset as the training set. It remains questionable to generalise our findings. To overcome this shortcoming, an external dataset from another source is required for external validation [29]. Furthermore, the willingness of receiving KR varies among different ethnic races but given that our study focused on exploring factors that influence the need for KR in terms of clinical, demographic, and lifestyle factors, race was excluded from our analysis [44]. Besides, ethnicity can also play an important role in the likelihood of OA occurrence and progression. For instance, it is less prevalent for Caucasian women to develop KOA than Chinese women [45]. Given that the majority of subjects were Caucasians while African American and Asian ethnicity constituted a minor population in the OAI dataset, this prompts the need of further investigation into the generalizability of our model in other ethnic groups such as Chinese population.

### Conclusion

To conclude, a KR survival model with good discrimination power was developed, empowering stratification of KOA patients with a three-tier KR risk scoring system using self-administrable factors. It is envisioned to decipher modifiable risk factors to facilitate individualised healthcare for the betterment of primary and secondary KOA prevention.

## Author contributions

HL, LC, PC and CW conceived the study. HL and LC collected data. All authors contributed to the writing of the manuscript and approved the final version.

## Role of the funding source

This work is supported by the Hong Kong Polytechnic University Shen Zhen Research Institute “百城百園” scheme, Research Institute of Smart Ageing, and Hong Kong Innovation Technology Fund for Better Living (ITB/FBL/B046/22/S).

## Declaration of competing interest

The authors have no relevant competing interests to disclose.
